# Reflectance confocal microscopy features of *BRAF* V600E mutated thin melanomas detected by immunohistochemistry

**DOI:** 10.1371/journal.pone.0179745

**Published:** 2017-06-29

**Authors:** Ana Claudia Urvanegia, Juliana Casagrande Tavoloni Braga, Danielle Shitara, Jose Humberto Fregnani, Jose Ivanildo Neves, Clovis Antonio Pinto, Ashfaq A. Marghoob, Joao Pedreira Duprat, Gisele Gargantini Rezze

**Affiliations:** 1 Cutaneous Oncology Department, AC Camargo Cancer Center, São Paulo, Brazil; 2 Dermaimage Medical Associates, São Paulo, Brazil; 3 Barretos Cancer Hospital, Teaching and Research Institute, Barretos, São Paulo, Brazil; 4 Anatomy Pathology Department, AC Camargo Cancer Center, São Paulo, Brazil; 5 Dermatology Service, Memorial Sloan Kettering Skin Cancer Center, New York, United States of America; University of Queensland Diamantina Institute, AUSTRALIA

## Abstract

The classification of melanoma into four histological subtypes has been questioned regarding its clinical validity in providing relevant information for treatment for metastatic tumors. Specific genetic alterations are associated with particular clinical and histopathological features, suggesting that these could be helpful in refining existing melanoma classification schemes. We analyzed *BRAF* V600E mutated melanomas to explore the Reflectance confocal microscopy (RCM) utility as a screening aid in the evaluation of the most appropriate patients for genetic testing. Thus, 32 melanomas were assessed regarding their *BRAF* V600E mutational status. Experts blinded to dermoscopic images and V600E immunohistochemistry results evaluated RCM images regarding previously described melanoma features. *BRAF* positive melanomas were related to younger age (p = 0.035), invasive melanomas (p = 0.03) and to the presence of hiporreflective cells (p = 0.02), epidermal nests (p = 0.02), dermal-epidermal junction nests (p = 0.05), edged papillae (p = 0.05), and bright dots (p = 0.05), and to absence of junctional thickening due to isolated cells (p = 0.01) and meshwork (p = 0.02). This study can not characterize other mutations in the *BRAF*, because the immunohistochemistry is specific to the type V600E. The findings should encourage the genetic evaluation of *BRAF* mutation. This study highlights the potential of RCM as a supplementary tool in the screening of *BRAF-*mutated melanomas.

## Introduction

The fact that melanomas can present with varied morphologies and biologic behaviors including differences in rate of growth, anatomical location and propensity to metastasize has been known for quite some time. Initial attempts at subtyping melanomas based on their clinical and histopathology characteristics lead to the classification of these tumors into superficial spreading, nodular, acro-lentiginous, and lentigo maligna, among others. Some have tried to simplify this classification based on the degree of UV exposure into melanomas on chronic sun damaged skin, melanomas on intermittent sun exposed skin and melanomas on UV protected sites [1]. However, evidence is now starting to emerge that the underpinnings responsible for the morphology and biology of melanoma may primarily be due to their molecular profile (gene expression profile) [2]. It has been shown that approximately 50% of melanomas have a *BRAF*V600 mutation [3]. Few recent studies have sought to correlate *BRAF* mutational status with features observed via non-invasive evaluation techniques, such as dermoscopy [4,5,6]. Researches have noted that *BRAF* mutated melanomas tend to be of the superficial spreading type and often reveal peppering on dermoscopy [5]. Ruini et al. used Reflectance Confocal Microscopy (RCM) to evaluate 8 melanomas and suggested that RCM may provide more specific information on the cytoarchitectural structure of *BRAF* mutated melanomas [7]. Our present study was undertaken to determine whether the RCM characteristics of the *BRAF* V600E mutated melanomas differ from the *BRAF* wild type melanomas.

## Material and methods

This study was approved by the Institutional Review Board of A C Camargo Cancer Center, São Paulo, Brazil and it was registered under no. 1685/12.

### Subjects

This retrospective cohort study included 32 consecutively diagnosed melanomas; all of which were imaged with RCM prior to biopsy. Only thin melanomas were included as RCM is not able to reach deeper skin layers. Each of the 32 melanomas came from a different patient and all cases were diagnosed between 2011 and 2013. Informed consent was not necessary because the data were analyzed anonymously.

### Routine histopathology

The histopathology slides were stained with hematoxylin-eosin and the pathologist (C.P.) was required to comment on the following parameters according to the Pathology Department’s protocol: histological subtype, growth pattern, Breslow thickness, Clark's level, peritumoral inflammatory infiltrate, intratumoral inflammatory infiltrate, regression, lymphatic invasion, vascular invasion, perineural invasion, existence of associated nevus, microscopic satellites, and mitotic rate. Histopathology images were obtained of all cases using ScanScope Digital Slide Scanner (Aperio, Vista, CA, USA).

### *BRAF* immunohistochemistry

The archived specimens were retrieved and processed by deparaffinization followed by antigen retrieval and dilution using the anti-VE1 antibody, which was performed on Ventana Benchmark XT immunostainer (Roche Diagnostics, Burges Hill—UK), according to specified standard protocol. OptiView DAB IHC Detection Kit (Roche, Burges Hill-UK) was used to detect V600E *BRAF* protein expression. The slides were stained with anti-V600E *BRAF* (VE1) Mouse Monoclonal Primary Antibody (Roche, Burges Hill-UK). Positive control with *BRAF* V600E mutated colorectal adenocarcinoma was used for procedure standardization. The omission of this antibody in tissue known to be positive for the *BRAF* V600E mutation served as the negative control. All immunohistochemistry stained slides were evaluated by the same pathologist (PCA). Immunostaining was interpreted as positive or negative according to criteria proposed by Capper et al. [8] and Long et al. [9], VE1 antibody staining was considered positive if tumor cells showed unambiguous cytoplasmic staining. The staining reaction was considered negative if there was no evidence of staining or if there was only weak, focal staining of isolated cells.

Molecular biology testing is still the gold standard method for *BRAF* V600E status assessment. However, a plenty of studies have shown that VE1 antibody staining is a less expensive approach to rapidly evaluate the *BRAF* V600E mutation in melanomas [10].

**Fig 1:**
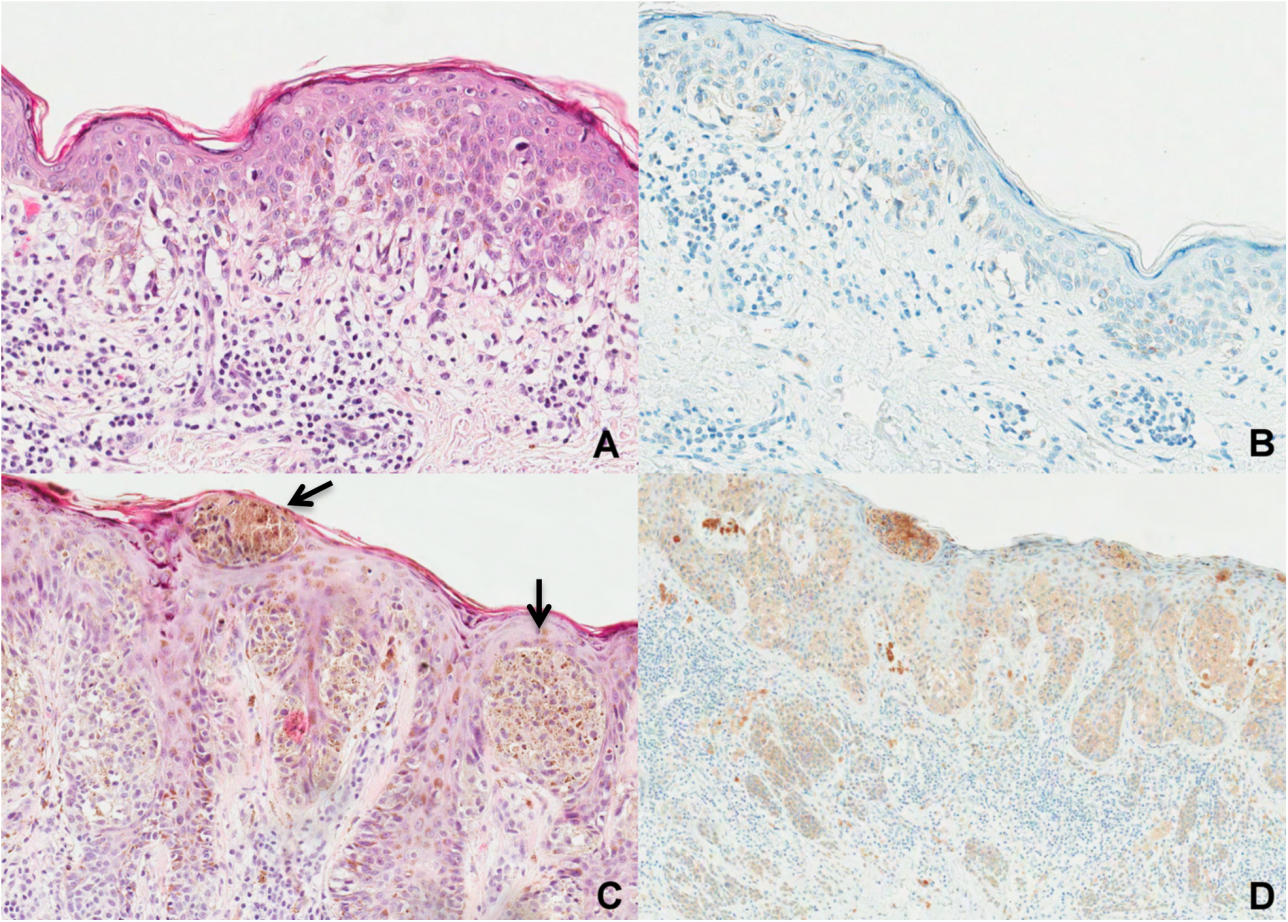
Tumor tissues with hematoxylin-eosin staining and with immunohistochemistry reaction for anti-VE1 antibody. (A) Histopathological features of *in situ* superficial spreading melanoma, exhibiting mainly scattered melanocytes along the basal layer and only few pagetoid cells spreading in the epidermis (H&E staining, original magnification 400x) (B) *BRAF* V600E IHC negative case characterized by complete lack of tumor cell immunostaining (original magnification 400x)—A and B corresponding to the case in Fig 2 (2A). (C) Histopathological features of superficial spreading melanoma, 1 mm in Breslow thickness showing a predominantly nested pattern of large intraepidermal and junctional nests (arrows) (H&E staining, original magnification 400x). (D) *BRAF* V600E cytoplasmic immunostaining positive (original magnification 200x)—C and D corresponding to the case in Fig 2D.

All study lesions proved to be relatively thin invasive melanomas or *in situ* melanomas. Hence the degree of *BRAF* staining in the *BRAF* mutated tumors was mostly weak to moderate and distributed focally or multifocally (Fig 1).

### Instruments

RCM images were acquired using the near-infrared reflectance confocal laser microscope (Vivascope 1500; Caliber I.D., Rochester, NY, USA). The RCM scanning technique and the acquisition of RCM images was the same as published by Pellacani et al. [11]. RCM “mosaic images”, ranging in size from 4x4 to 8x8 mm, were captured in all cases ("Vivablock"). A minimum of three mosaics was obtained for each lesion with at least one mosaic acquired at the superficial epidermal layer, one at dermal-epidermal junction (DEJ) and one at the papillary dermis. In addition, focal areas of interest were imaged via the “stack” method, which captures images of the area of interest by acquiring sequential images from the stratum corneum up to papillary dermis.

### Parameter evaluation

RCM: Two dermatologists with experience in reading RCM images (TBJC and RGG) independently evaluated all of the RCM images blinded to both dermoscopic and histopathological features. The findings were categorized in a binary fashion for the following parameters [12,13,14].

1. Epidermal layer: presence or absence of atypical honeycomb pattern, atypical cobblestone pattern, pagetoid pattern and hyporreflective cells. If within any 1x1mm field of view there were more then 10 pagetoid cells per mm^2^ we considered this parameter to be present and if ≤ 10 pagetoid cells it was considered as absent. Hyporreflective cells were considered present if more than 5 cells were seen in any high power field (1x1).
2. Dermal-epidermal junction (DEJ): presence or absence of junctional nests, edged papillae, non-edged papillae, non-visible papillae, sheet-like structures, mitochondria-like structures, meshwork pattern and junctional thickening (enlargement of the inter-papillary spaces by bright sparse cells or in aggregates <5 cells).
3. Superficial dermis: presence or absence of bright dots, sparse plump cells, aggregated plump cells, reticulated collagen, and collagen in bundles.

### Statistical analysis

The results were tabulated and the frequency distributions, standard deviations, and range of measurements calculated. The Mann -Whitney test was used to compare the mean age as a function of *BRAF* V600E expression. The Fisher's Exact Test was used to evaluate the association between the expression of *BRAF* V600E and the presence or absence of: growth phase (radial or vertical), hyporreflective cells, epidermal nests, dermal-epidermal junction (DEJ) nests, edged papillae, nonedged papillae, junctional thickening, meshwork pattern and bright dots.

Multiple logistic regression was employed to identify the independent RCM factors predictive of positive *BRAF* V600E mutation status. All RCM variables seen in *BRAF* V600E mutated and *BRAF* wild type melanomas were compared and any variable that was found to be different between these two groups, with p value of less than 0.20, in univariate analysis were selected for a stepwise forward selection model and only variables with a p value less than or equal to 0.05 remained in the final multiple logistic model. The predictive value of the final model was assessed by calculating the area under the ROC curve (AUC).

Microsoft Excel 2007 (www.microsoft.com) and the IBM SPSS version 22.0.0.0 were used for all analyses.

## Results

A total of 32 melanomas from 16 women and 16 men with ages ranging between 28 and 85 years were evaluated. Four melanomas (12.5%) were located on the head and neck area, 13 (40.6%) on the torso, 12 (37.5%) on the arm and 3 on the leg (Table 1).

**Table 1:** Clinical variables and *BRAF* V600E mutation.

		*BRAF* V600E			
Variable	Category	negative^a^	positive^b^	Total	P value
Gender	Male	13 (56.5%)	3 (33.3%)	16	0.4
	Female	10 (43.4%)	6 (66.7%)	16	
*Age	Mean (SD)	58.6 (14.3)	58.1 (14.6)		**0.02**
*Age < 50years old	Mean (SD)	56.7 (14.1)	58.1 (14.6)		**0.03**
Topography	Head and neck	3 (75.0%)	1 (25.0%)	4	0.9
	Superior limb	8 (66.7%)	4 (33.3%)	12	
	Trunk	10 (76.9%)	3 (23.1%)	13	
	Inferior limb	2 (66.7%)	1(33.3%)	3	

*Mann Whitney Test

a. Negative: absence of staining

b. Positive: any positive staining (focal or multifocal).

Histopathology variables were assessed and are summarized in Table 2.

**Table 2:** Histological variables and *BRAF* V600E mutation.

		*BRAF* V600E			
Variable	Category	negative^a^	positive^b^	Total	P value
Histological subtype	S. Spreading	19 (67.9%)	9 (32.1%)	28	1.0
	L. Maligna	2 (100%)	0	2	
	not informed	2 (100%)	0	2	
**Growth	In situ	17 (94.4%)	1 (5.6%)	18	**0.03**
	Radial	3 (42.9%)	4 (57.1%)	7	
	Vertical	3 (42.9%)	4 (57.1%)	7	
Ulceration	Absent	6 (42.9%)	8 (57.1%)	14	+
Clark	II	3 (42.9%)	4 (57.1%)	7	1.0
	III	3 (42.9%)	4 (57.1%)	7	
Breslow	Mean (SD)	0.67 (0.23)	0.64 (0.25)		0.6
Mitotic Index mm^2^	Mean (SD)	0.40 (0.92)	0.37 (0.89)		0.6
Peritumoral Invasion	Present	6 (46.2%)	7 (53.8%)	13	1.0
	Absent	0	1 (100%)	1	
Intratumoral Invasion	Present	2 (28.6%)	5 (71.4%)	7	0.6
	Absent	4 (57.1%)	3 (42.9%)	7	
Regression	Absent	6 (42.9%)	8 (57.1%)	14	+
Lymphatic Invasion	Absent	6 (42.9%)	8 (57.1%)	14	+
Vascular Invasion	Absent	6 (42.9%)	8 (57.1%)	14	+
Perineural Invasion	Absent	6 (42.9%)	8 (57.1%)	14	+
Previous nevus	Present	6 (66.7%)	3 (33.3%)	9	0.7
	Absent	17 (73.9)	6 (26.1%)	23	
Microscopic Satelitosis	Absent	6 (42.9%)	8 (57.1%)	14	+
Margins	Free	20 (69.0%)	9 (31.0%)	29	0.5
	Positive	3 (100%)	0	3	

**Fisher’s Exact Test

a. Negative: absence of staining

b. Positive: any positive staining (focal or multifocal).

Superficial spreading melanoma was the most common histological subtype, comprising 87.5% (n = 28) of the cases and they were located on the torso (n = 11), limbs (n = 13) and head and neck (n = 4). Two cases were classified as lentigo maligna melanomas and they were located on the upper trunk and upper limb. In two cases the histological subtype could not be determined, since there was an overlapping of features (superficial spreading melanoma and lentigo maligna). Most melanomas were in situ (18/32; 56.3%) and were located on the head&neck (n = 1), torso (n = 9), upper limbs (n = 7) and lower limbs (n = 1). The invasive melanomas had a median Breslow thickness of 0.66mm and most had a Clark level between II and III. Peritumoral and intratumoral infiltrates were seen in 92.7% and 50% of melanomas, respectively. No ulceration, regression, satellitosis, perineural invasion, vascular invasion, nor lymphatic invasion was observed. Nine melanomas (9/32; 28.1%) were found to have an associated melanocytic nevus.

### RCM analysis

RCM images revealed that all 32 melanomas had an atypical honeycomb pattern, non-edged papillae and reticulated collagen. Other frequent features observed were atypical cobblestone (93.8%), sparse plump cells (90.4%), bright dots (62.5%), pagetoid cells (62.5%), edged papillae (62.5%), dermal-epidermal junction nests (59.4%) and sheet-like structures (53.1%). Most epidermal nests observed on RCM were of the dense variety. The distribution of RCM parameters is described further in Table 3.

**Table 3:** Confocal microscopy characteristics and *BRAF* V600E mutation.

		*BRAF* V600E			
Variable	Category	negative^a^	positive^b^	Total	P value
Atypical Honeycomb	Present	23 (71.9%)	9 (28.1%)	32	+
Atypical Cobblestone	Present	22 (73.3%)	8 (26.7%)	30	0.5
	Absent	1 (50.0%)	1 (50.0%)	2	
Pagetoid cells	Present	12 (60.0%)	8 (40.0%)	20	0.1
	Absent	11 (91.7%)	1 (8.3%)	12	
**Hyporreflective cells	Present	3 (37.5%)	5 (62.5%)	8	**0.02**
	Absent	20 (83.3%)	4 (16.7%)	24	
**Epidermal nests	Present	7 (50.0%)	7(50.0%)	14	**0.02**
	Absent	16 (88.9%)	2 (11.1%)	18	
**DEJ nests	Present	11 (57.9%)	8 (42.1%)	19	**0.05**
	Absent	12 (92.3%)	1 (7.7%)	13	
**Edged papillae	Present	17 (85.0%)	3 (15.0%)	20	**0.05**
	Absent	6 (50.0%)	6 (50.0%)	12	
Nonedged papillae	Present	23 (71.9%)	9 (28.1%)	32	+
**Abscence of papillae	Present	1 (25.0%)	3 (75.0%)	4	**0.06**
	Absent	22 (78.6%)	6 (21.4%)	28	
Sheet-like Structure	Present	12 (70.6%)	5 (29.4%)	17	1.0
	Absent	11 (73.3%)	4 (26.7%)	15	
**Meshwork	Present	16 (88.9%)	2 (11.1%)	18	**0.02**
	Absent	7 (50.0%)	7 (50.0%)	14	
Mitochondria	Present	4 (80.0%)	1 (20.0%)	5	1.0
	Absent	19 (70.4%)	8 (29.6%)	27	
	Absent	20 (69.0%)	9 (31.0%)	29	
**Bright dots	Present	17 (85.0%)	3 (15.0%)	20	**0.05**
	Absent	6 (50.0%)	6 (50.0%)	12	
Sparse Plump cells	Present	22 (75.9%)	7 (24.1%)	29	0.2
	Absent	1 (33.3%)	2 (66.7%)	3	
Agregated Plump cells	Present	8 (72.7%)	3 (27.3%)	11	1.0
	Absent	15 (71.4%)	6 (28.6%)	21	
Reticulated Collagen	Present	23 (71.9%)	9 (28.1%)	32	+
Collagen Bundles	Present	5 (83.3%)	1 (16.7%)	6	0.6
	Absent	18 (69.2%)	8 (30.8%)	26	

**Fisher’s Exact Test

a. Negative: absence of staining

b. Positive: any positive staining (focal or multifocal).

### *BRAF* analysis

**Fig 2:**
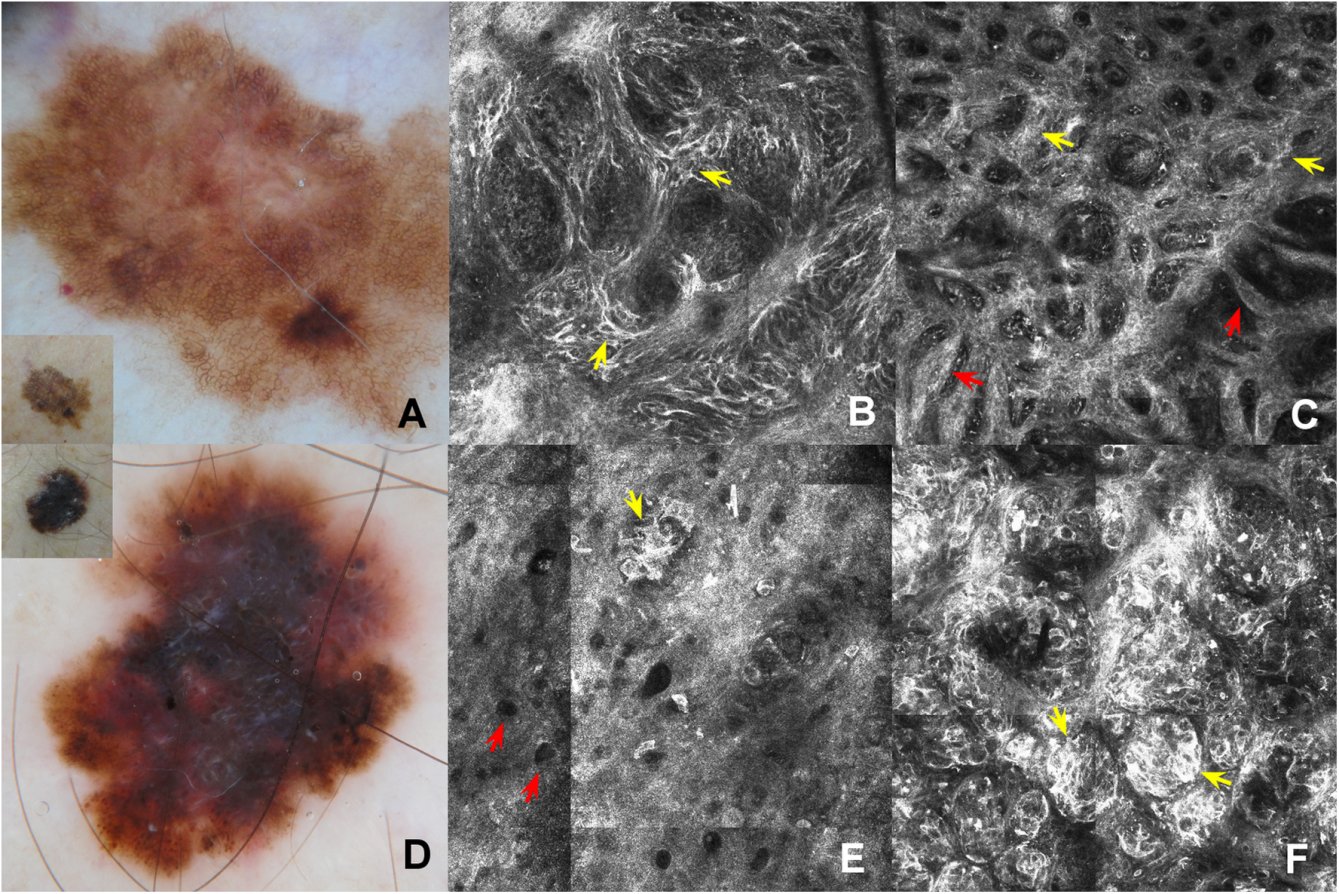
Comparison between findings of dermatoscopy and RCM. (A) Dermoscopy of *BRAF* V600E negative *in situ* superficial spreading melanoma: broadened pigmented network (the inset corresponds to clinical image). (B) RCM mosaic image (1.5x1.5 mm) in DEJ shows junctional thickening due to isolated atypical cells (yellow arrows). (C) RCM mosaic image (1.5x1.5 mm) in DEJ showing non-edged papillae separated by loosely thick interpapillary spaces (yellow arrows) and meshwork pattern (red arrows). (D) Dermoscopy of *BRAF* V600E mutated superficial spreading melanoma, 1 mm in Breslow thickness): multicomponent pattern (the inset corresponds to clinical image). (E) RCM mosaic image (0.75x0.75 mm) at the level of the epidermis shows hyporeflective pagetoid cells (red arrows) and epidermal nests (yellow arrow). (F) RCM mosaic image (0.75x0.75 mm) at the level of the DEJ shows dermal-epidermal nests (yellow arrows).

Nine of 32 melanomas stained positive for the *BRAF* V600E mutation (28.1%; Figs 1 and 2).

**Fig 3:**
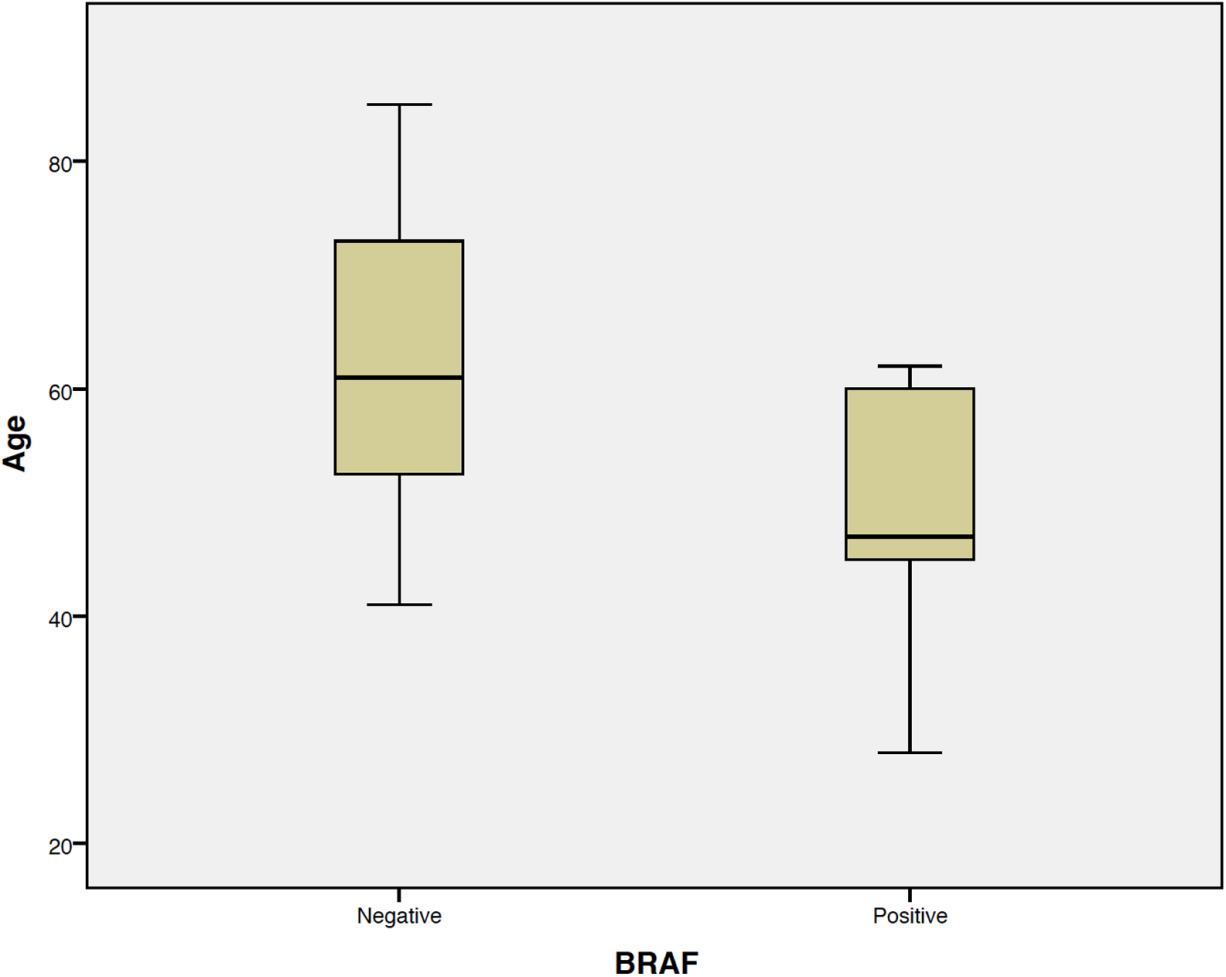
Graphic age x *BRAF* V600E.

Patients with *BRAF* positive melanomas were significantly younger (dichotomized into those ≤50 vs >50 years of age) as compared to patients with *BRAF* wild type melanoma (p = 0.035) (Fig 3).

Melanomas that were *BRAF* mutated were more likely to be invasive (p = 0.03). No difference was observed in *BRAF* mutated vs *BRAF* wild type melanomas vis-a-vis patient gender, primary site of tumor, association with melanocytic nevus, nor any other histopathological parameter (Table 2).

Differences in RCM parameters between *BRAF* mutated and wild type melanoma was observed. *BRAF* positive melanomas were more likely to reveal hyporreflective cells (p = 0.02; Fig 2E), epidermal nests (p = 0.02), dermal-epidermal junction nests (p = 0.05), edged papillae (p = 0.05), and bright dots (p = 0.05; Table 3). In addition, *BRAF* mutated melanomas were less likely to reveal junctional thickening (p = 0.01) and a meshwork pattern (p = 0.02; Table 4).

**Table 4:** Clinical and histological characteristics of *BRAF* V600E mutated melanomas.

**Case**	**Gender**	**Age**	**Local**	Histological Type	Growth	Clark	Breslow	Mitotic index mm^2^	Peritumoral invasion*	Intratumoral Invasion*	Pre-existing. Nevus*	HRC (1)*	EN (2)*	DEJN (3)*	EP (4)*	AP (5)*	N (6)*	MS (7)*	BD (8)*
1	Female	45	Upper limbs	SS	In situ						0	1	0	1	1	1	1	0	0
2	Male	60	Upper limbs	SS	Radial	II	0.26	0	0	0	1	0	0	0	0	0	1	1	1
3	Female	47	Trunk	SS	Radial	III	0.79	1.25	1	0	1	1	1	1	0	0	1	0	0
4	Female	42	Head and neck	SS	Vertical	II	0.52	0	1	1	0	1	1	1	0	1	1	0	0
5	Male	61	Lower limbs	SS	Vertical	III	1	0	1	1	0	1	1	1	0	0	0	0	0
6	Female	28	Trunk	SS	Vertical	III	1	0	1	1	0	0	1	1	0	1	0	0	0
7	Female	62	Trunk	SS	Vertical	II	0.5	0	1	0	1	0	1	1	1	0	1	0	0
8	Male	47	Upper limbs	SS	Radial	III	0.71	0	1	1	0	1	1	1	1	0	1	1	1
9	Female	45	Upper limbs	SS	Radial	II	0.7	0	1	1	0	0	1	1	0	0	1	0	1

^(1)^Hyporreflective cells

^(2)^Epidermal nests

^(3)^DEJ nests

^(4)^Edged papillae

^(5)^Abscence papillae

^(6)^Junctional thickening cell nests

^(7)^Meshwork

^(8)^Bright dots.

^*^0 means absence of parameter and 1 means presence of parameter.

SS: Superficial Spreading

Multiple logistic regression (Table 5) identified the following independent RCM features predictive of *BRAF* V600E positivity: absence of meshwork pattern (OR = 14.3; 95%CI: 1.3–156.8) and absence of junctional thickening (OR = 16.7; 95% CI: 1.6–175.0).

**Table 5:** Multivariate analysis for the identification of independent predictive factors for positive BRAF V600E.

Variable	n	OR	95%CI
**Meshwork**			
Absent	14	14.3	1.3–156.8
Present	18	1.0	Reference
**Junctional thickening cell agregate**			
Absent	10	16.7	1.6–175.0
Present	22	1.0	Reference

OR: Odds ratio

95%CI: 95% Confidence interval

Number of events: 9 (*BRAF* V600E +)

The distribution of cases as a function of *BRAF* status and the number of cumulative predictive factors for *BRAF* V600E positivity are depicted in Table 6.

**Table 6:** Distribution of cases according to BRAF V600E status and number of cumulative predictive factors.

Number of cumulative predictive factors (*)	*BRAF* V600E			
	Negative		Positive	
	N	(%)	N	(%)
None	13	(100.0)	0	(0.0)
One factor	9	(64.3)	5	(35.7)
Two factors	1	(20.0)	4	(80.0)

Fisher’s exact test: P = 0.002; Area under the curve (AUC): 0.86 (95%CI: 0.73–0.99).

(*) Predictive factors: absence of meshwork and absence of junctional thickening cell agregate.

**Fig 4:**
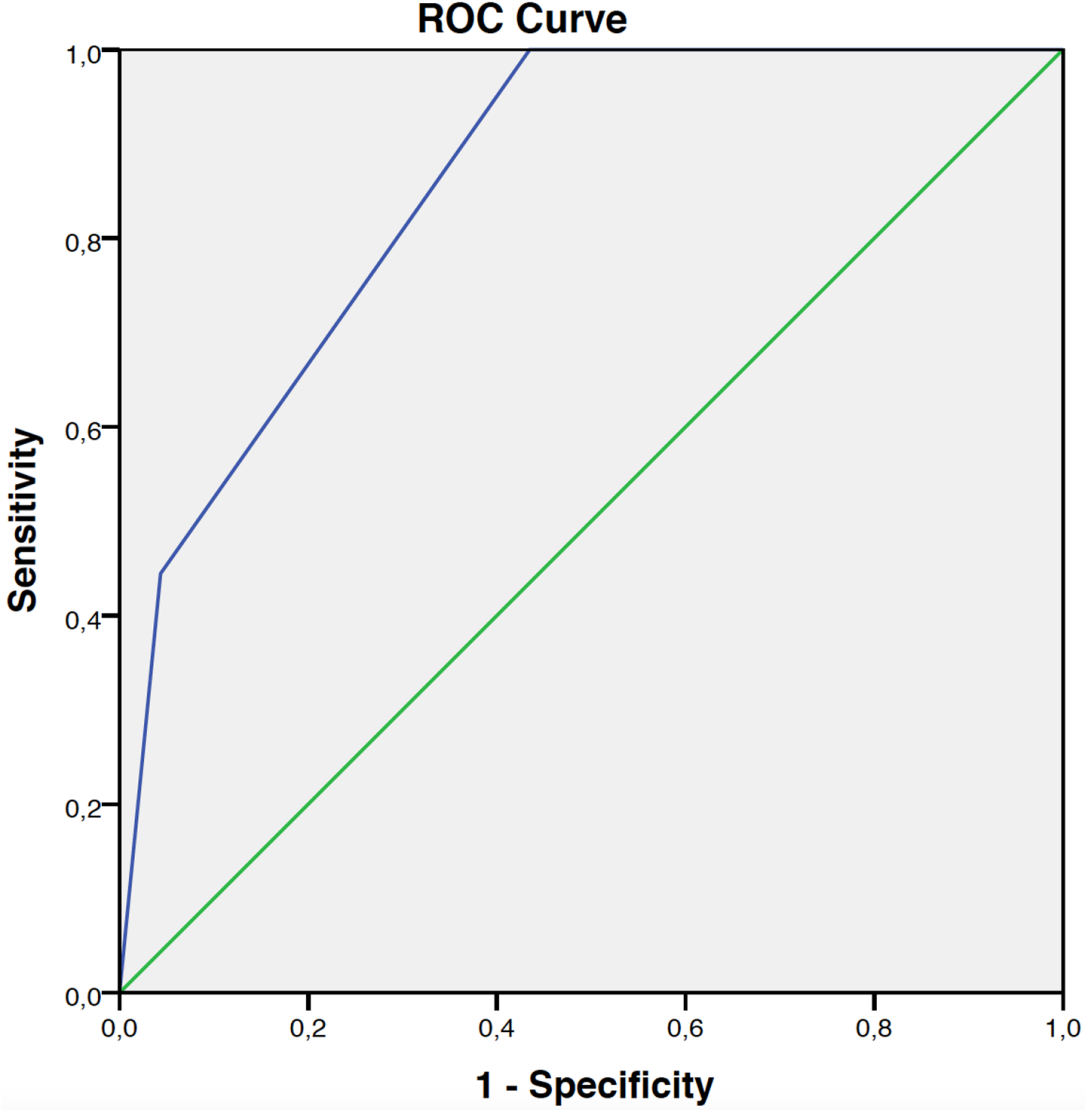
ROC curve of positivity for *BRAF* V600E.

There was a statistically significant difference in *BRAF* positivity as a function of the number of cumulative factors (p = 0.002) present, ranging from zero up to 80%. This model had an AUC equal to 0.86 (95% CI: 0.73–0.99), as depicted in Fig 4.

## Discussion

The discovery that melanomas could be sub-classified based on their mutation profile provided the basis for the molecular classification of cutaneous melanoma [15]. While many mutations have been identified in melanoma, the most common is the *BRAF* V600E mutation, which is seen in approximately 50% of cutaneous melanomas [9,16,17] Studies have shown that *BRAF* mutated melanomas tend to develop in younger individuals as compared to *BRAF* wild type melanomas [18,2]. Indeed, 66.7% (6 of 9) of the patients with *BRAF*-mutated melanomas in our cohort were younger than 50 years of age. In contrast, 86.9% of the patients with *BRAF wild type* melanomas were older than 50; similar observations were also made by Menzies et al. [18]. Approximately one third of the *BRAF* mutated melanomas in our cohort were found to have an associated nevus in the histologic examination. Similar observations were made by Shitara et al. [19], who noted that 38.3% (23/60) of their V600E-mutated melanomas had an associated nevus that was also V600E-mutated.

RCM enables clinicians to visualize subsurface skin structures on the cellular level *in vivo*. The features seen on RCM correlate well with features seen on routine histological examination [20,21,22]. One previous study evaluated six *BRAF* V600E mutated primary melanomas and 2 metastasis [7] with RCM and found them to reveal pleomorphic pagetoid cells, disarrangement of the dermal-epidermal junction, discohesive junctional nests and bright particles at the DEJ. In our cohort, the presence of bright dots, which correlates with inflammation (leukocyte infiltration), was the only parameter found in common with Ruini et al.’s study being significantly associated with *BRAF* V600E status.

Since the presence of both bright dendritic and roundish cells in the spinous layer correlate with the presence of pagetoid cells on histopathology [23], we elected not to differentiate between these two subtypes of pagetoid cells seen on RCM. While Viros et al. observed on histopathology that increased upward scatter of intraepidermal melanocytes was associated with *BRAF* mutations [24], we did not find the same on RCM imaging. However, we did find that the presence of ‘hyporeflective pagetoid cells’ (HPCs), described as round dark structures (similar to ‘holes’) within the epidermis [25], was significantly correlated with *BRAF* V600E mutation. Of interest, these hyporeflective cells have been described in hypomelanotic melanomas, Paget's disease [26], and rarely in pigmented melanomas [24].

One of the novel findings of our study was that not only the presence of dermal-epidermal nest but also the presence of intraepidermal nests are associated with *BRAF* V600E mutations. This finding is consistent with Viros et al.’s histopathology study showing that intraepidermal melanocytes arranged in nests were positively associated with *BRAF*-mutation status [24]. Since *BRAF* V600E mutations are commonly seen in dermal nevi, it is tempting to speculate whether *BRAF* mutations can directly lead to nest formation [27,28,6]. It is interesting to note that while many melanomas reveal sparse nests, the nests in our *BRAF* mutated melanomas were dense; similar to nests seen in intradermal nevi, most of which also happen to harbor *BRAF* V600E mutations [29], No cerebriform nests were observed, most probably because the melanomas in our cohort were thin tumors.

Recently an attempt has been made to group melanomas based on their RCM features. Four types of melanomas have been identified: dendritic cell melanomas, round cell melanomas, dermal nest melanomas, and combined type melanomas. Melanomas with a meshwork pattern have been associated with both dendritic and round cell melanomas [30]. While dendritic cell melanomas were associated with the presence of thin meshwork pattern and dendritic cells, round cell melanomas were associated with the presence of large round cells distributed in a pagetoid fashion and with nest formation. This latter type melanoma was found to be associated with *BRAF* V600E mutations. Patients with this type of melanoma tend to be younger, have multiple nevi and rarely develop melanoma on chronic sun exposed skin. In contrast, dendritic cell melanomas tend to reveal junctional thickening resulting from isolated atypical cells without nest formation. These melanomas tend to be *BRAF* wild type [30]. Indeed, after multivariate analysis, the most important predictive factors for *BRAF* V600E positivity were the absence of meshwork and/or absence of junctional thickening. In other words, if both parameters are absent there is a high likelihood that the melanoma will harbor the *BRAF* V600E mutation.

The findings of the present study reveals that *BRAF* V600E mutated melanomas tend to have epidermal and DEJ nests, are of the superficial spreading type, occur at a younger age, and are located on intermittently sun exposed areas. In contrast, *BRAF wild type* melanomas develop at an older age, reveal a thin meshwork pattern and junctional thickening. The aforementioned supports the existence of different pathways to melanomas, leading to different subsets of melanomas, each depicting particular clinical, dermoscopic and RCM features (Table 7).

**Table 7:** Clinical, dermoscopic, confocal microscopy and histopathological features of positive and negative BRAF V600E melanomas.

*BRAF* V600E	Clinic	Dermoscopy	Confocal microscopy	Histopathology
POSITIVE	Age < 50 (*p* = 0,022);	Globular pattern with irregular globules; Blotches	Hyporeflective cells in epidermis (*p* = 0,21); Epidermal nests (*p* = 0,019); Junctional nests (*p* = 0,035);	Radial growth (*p* = 0,003); Vertical growth (*p* = 0,003)
NEGATIVE	Age > 50 (*p* = 0,022);	Atypical network; Sparse irregular globules; Radial projections; Irregular hypopigmentation; Veil; Peppering	Edged papillae (*p* = 0,038); Junctional thickening cell aggregates (*p* = 0,012); Meshwork (*p* = 0,019);	*In situ* (*p* = 0,003)

Melanomas occurring at a younger age, on intermittently sun-exposed areas, with tendency to nest formation are clearly different from melanomas in older patients, on chronically sun exposed areas, with histopathological tendency of more lentiginous proliferation.

The present study highlights the potential of RCM to be used as a supplementary tool in the screening for *BRAF-*mutated melanomas. The findings of the presence of intraepidermal nests, DEJ nest, bright dots, in the absence of junctional thickening and absence of meshwork is highly suggestive that the melanoma in question will harbor a *BRAF* V600E mutation.

## References

[1] Whiteman DC, Watt P, Purdie DM, Hughes MC, Hayward NK, Green AC. Melanocytic nevi, solar keratoses, and divergent pathways to cutaneous melanoma. J Natl Cancer Inst. 2003;95:806–12. . 12783935 10.1093/jnci/95.11.806

[2] Whiteman DC, Pavan WJ, Bastian BC. The melanomas: a synthesis of epidemiological, clinical, histopathological, genetic, and biological aspects, supporting distinct subtypes, causal pathways, and cells of origin. Pigment Cell Melanoma Res. 2011;24:879–97. doi: 10.1111/j.1755-148X.2011.00880.x . 21707960 10.1111/j.1755-148X.2011.00880.x PMC3395885

[3] Davies H, Bignell GR, Cox C, Stephens P, Edkins S, Clegg S, et al Mutations of the BRAF gene in human cancer. Nature. 2002;417:949–54. doi: 10.1038/nature00766 12068308 10.1038/nature00766

[4] Ponti G, Pellacani G, Tomasi A, Loschi P, Luppi G, Gelsomino F, et al Molecular targeted approaches for advanced BRAF V600, N-RAS, c-KIT, and GNAQ melanomas. Dis Markers. 2014;671283. doi: 10.1155/2014/671283. Erratum in: Dis Markers. 2014;2014:246751 . 24591764 10.1155/2014/671283 PMC3925612

[5] Pozzobon FC, Puig-Butillé JA, González-Alvarez T, Carrera C, Aguilera P, Alos L, et al Dermoscopic criteria associated with BRAF and NRAS mutation status in primary cutaneous melanoma. Br J Dermatol. 2014;171:754–9. doi: 10.1111/bjd.13069 . 24749938 10.1111/bjd.13069

[6] Zalaudek I, Guelly C, Pellacani G, Hofmann-Wellenhof R, Trajanoski S, Kittler H, et al The dermoscopical and histopathological patterns of nevi correlate with the frequency of BRAF mutations. J Invest Dermatol. 2011;131:542–5. doi: 10.1038/jid.2010.332 . 21068756 10.1038/jid.2010.332

[7] Ruini C, Manfredini M, Pellacani G, Mandel VD, Tomasi A, Ponti G. Confocal microscopy characterization of BRAFV600E mutated melanomas. Melanoma Res. 2015;25:367–71. doi: 10.1097/CMR.0000000000000147 . 26134486 10.1097/CMR.0000000000000147

[8] Capper D, Preusser M, Habel A, Sahm F, Ackermann U, Schindler G, et al Assessment of BRAF V600E mutation status by immunohistochemistry with a mutation-specific monoclonal antibody. Acta Neuropathol. 2011;122:11–9. doi: 10.1007/s00401-011-0841-z . 21638088 10.1007/s00401-011-0841-z

[9] Long GV, Wilmott JS, Capper D, Preusser M, Zhang YE, Thompson JF, et al Immunohistochemistry is highly sensitive and specific for the detection of V600E BRAF mutation in melanoma. Am J Surg Pathol. 2013;37:61–5. doi: 10.1097/PAS.0b013e31826485c0 . 23026937 10.1097/PAS.0b013e31826485c0

[10] Long E, Ilie M, Lassalle S, Butori C, Poissonnet G, Washetine K, et al Why and how immunohistochemistry should now be used to screen for the BRAFV600E status in metastatic melanoma? The experience of a single institution (LCEP, Nice, France). J Eur Acad Dermatol Venereol. 2015;29:2436–43. doi: 10.1111/jdv.13332 . 26377147 10.1111/jdv.13332

[11] Pellacani G, Guitera P, Longo C, Avramidis M, Seidenari S, Menzies S. The impact of in vivo reflectance confocal microscopy for the diagnostic accuracy of melanoma and equivocal melanocytic lesions. J Invest Dermatol. 2007;127:2759–65. doi: 10.1038/sj.jid.5700993 17657243 10.1038/sj.jid.5700993

[12] Braga JC, Macedo MP, Pinto C, Duprat J, Begnami MD, Pellacani G, et al. Learning reflectance confocal microscopy of melanocytic skin lesions through histopathologic transversal sections. PLoS One. 2013;8:81205 doi: 10.1371/journal.pone.0081205 . 24339910 10.1371/journal.pone.0081205 PMC3855214

[13] Pellacani G, Farnetani F, Gonzalez S, Longo C, Cesinaro AM, Casari A, et al In vivo confocal microscopy for detection and grading of dysplastic nevi: a pilot study. J Am Acad Dermatol. 2012;66:109–21. doi: 10.1016/j.jaad.2011.05.017 . 21742408 10.1016/j.jaad.2011.05.017

[14] Rezze GG, Casagrande J. Atlas de microscopia confocal na dermatologia, 1st ed. São Paulo, SP: Lemar, 2015.

[15] Flaherty KT, Fisher DE. New strategies in metastatic melanoma: oncogene-defined taxonomy leads to therapeutic advances. Clin Cancer Res. 2011;17:4922–8. doi: 10.1158/1078-0432.CCR-10-2612 . 21670085 10.1158/1078-0432.CCR-10-2612

[16] Singh M, Lin J, Hocker TL, Tsao H. Genetics of melanoma tumorigenesis. Br J Dermatol. 2008;158:15–21. doi: 10.1111/j.1365-2133.2007.08316.x 18047516 10.1111/j.1365-2133.2007.08316.x

[17] Tschandl P, Berghoff AS, Preusser M, Burgstaller-Muehlbacher S, Pehamberger H, Okamoto I, et al NRAS and BRAF mutations in melanoma-associated nevi and uninvolved nevi. PLoS One. 2013;8:69639 doi: 10.1371/journal.pone.0069639 . 23861977 10.1371/journal.pone.0069639 PMC3704624

[18] Menzies AM, Haydu LE, Visintin L, Carlino MS, Howle JR, Thompson JF, et al Distinguishing clinicopathologic features of patients with V600E and V600K BRAF-mutant metastatic melanoma. Clin Cancer Res. 2012;18:3242–9. doi: 10.1158/1078-0432.CCR-12-0052 . 22535154 10.1158/1078-0432.CCR-12-0052

[19] Shitara D, Tell-Martí G, Badenas C, Enokihara MM, Alós L, Larque AB, et al Mutational status of naevus-associated melanomas. Br J Dermatol. 2015;173:671–80. doi: 10.1111/bjd.13829 . 25857817 10.1111/bjd.13829 PMC4583836

[20] Langley RG, Walsh N, Sutherland AE, Propperova I, Delaney L, Morris SF, et al The diagnostic accuracy of in vivo confocal scanning laser microscopy compared to dermoscopy of benign and malignant melanocytic lesions: a prospective study. Dermatology. 2007;215:365–72. doi: 10.1159/000109087 17912001 10.1159/000109087

[21] Pellacani G, Cesinaro AM, Seidenari S. In vivo confocal reflectance microscopy for the characterization of melanocytic nests and correlation with dermoscopy and histology. Br J Dermatol. 2005;152:384–6. doi: 10.1111/j.1365-2133.2005.06348.x 15727668 10.1111/j.1365-2133.2005.06348.x

[22] Zalaudek I, Leinweber B, Hofmann-Wellenhof R, Scope A, Marghoob AA, Ferrara G, et al The epidermal and dermal origin of melanocytic tumors: theoretical considerations based on epidemiologic, clinical, and histopathologic findings. Am J Dermatopathol. 2008;30:403–6. doi: 10.1097/DAD.0b013e3181734e9a . 18645318 10.1097/DAD.0b013e3181734e9a

[23] Longo C, Rito C, Beretti F, Cesinaro AM, Piñeiro-Maceira J, Seidenari S, et al De novo melanoma and melanoma arising from pre-existing nevus: in vivo morphologic differences as evaluated by confocal microscopy. J Am Acad Dermatol. 2011;65:604–14. doi: 10.1016/j.jaad.2010.10.035 . 21715047 10.1016/j.jaad.2010.10.035

[24] Viros A, Fridlyand J, Bauer J, Lasithiotakis K, Garbe C, Pinkel D, et al Improving melanoma classification by integrating genetic and morphologic features. PLoS Med. 2008;5:120 doi: 10.1371/journal.pmed.0050120 . 18532874 10.1371/journal.pmed.0050120 PMC2408611

[25] Losi A, Longo C, Cesinaro AM, Benati E, Witkowski A, Guitera P, et al Hyporeflective pagetoid cells: a new clue for amelanotic melanoma diagnosis by reflectance confocal microscopy. Br J Dermatol. 2014;171:48–54. doi: 10.1111/bjd.12781 . 24329036 10.1111/bjd.12781

[26] Pan ZY, Liang J, Zhang QA, Lin JR. Zheng ZZ. In vivo reflectance confocal microscopy of extramammary Paget disease: diagnostic evaluation and surgical management. J Am Acad Dermatol. 2012;66:47–53. doi: 10.1016/j.jaad.2010.09.722 . 21620517 10.1016/j.jaad.2010.09.722

[27] Gill M, Celebi JT. B-RAF and melanocytic neoplasia. J Am Acad Dermatol. 2005;53:108–14. doi: 10.1016/j.jaad.2005.04.013 15965430 10.1016/j.jaad.2005.04.013

[28] Marchetti MA, Kiuru MH, Busam KJ, Marghoob AA, Scope A, Dusza SW, et al Melanocytic naevi with globular and reticular dermoscopic patterns display distinct BRAF V600E expression profiles and histopathological patterns. Br J Dermatol. 2014;171:1060–5. doi: 10.1111/bjd.13260 . 25039578 10.1111/bjd.13260

[29] Benati E, Argenziano G, Kyrgidis A, Moscarella E, Ciardo S, Bassoli S, et al Melanoma and naevi with a globular pattern: confocal microscopy as an aid for diagnostic differentiation. Br J Dermatol. 2015;173:1232–8. doi: 10.1111/bjd.14049 . 26212145 10.1111/bjd.14049

[30] Pellacani G, De Pace B, Reggiani C, Cesinaro AM, Argenziano G, Zalaudek I, et al Distinct melanoma types based on reflectance confocal microscopy. Exp Dermatol. 2014;23:414–8. doi: 10.1111/exd.12417 . 24750486 10.1111/exd.12417

